# Prognostic factors for intraoperative detection of necrotizing fasciitis in severe soft tissue infections

**DOI:** 10.1371/journal.pone.0285048

**Published:** 2023-05-03

**Authors:** Thaer S. A. Abdalla, Rainer Grotelüschen, Ahmed S. A. Abdalla, Nathaniel Melling, Jakob R. Izbicki, Kai Bachmann

**Affiliations:** Department of General, Visceral and Thoracic Surgery, at the University Hospital Hamburg-Eppendorf, Hamburg, Germany; University of Pittsburgh Medical Center Pinnacle Health Medical Services, UNITED STATES

## Abstract

**Background:**

Necrotizing fasciitis (NF) is a rare but lethal soft-tissue infection. There is still a paucity of information regarding the diagnostic tools and therapeutic strategies for the treatment of this devastating disease. This study aims to identify important perioperative parameters related to necrotizing fasciitis and to assess their relevance in terms of identifying NF.

**Methods and material:**

We retrospectively analyzed patients who underwent surgical exploration for suspected necrotizing fasciitis at a tertiary referral center, to explore the clinical features and factors related to the presence of necrotizing fasciitis and mortality.

**Results:**

Between 2010 and 2017, 88 patients underwent surgical exploration for suspected NF. The infection occurred in the lower extremities in 48 patients, in the thoracocervical region in 18 patients, and the perineum and abdomen in 22 patients. Histological evidence of NF was present in 59 of 88 patients. NF was associated with a longer hospital stay and ICU stay (p = 0.05 and 0.019 respectively) compared to patients without NF. ROC analysis showed that only macroscopic fascial appearance could discriminate patients with histological evidence of NF. Moreover, multivariate logistic regression revealed, that liver failure (p = 0.019), sepsis (p = 0.011), positive Gram stain (p = 0.032), and macroscopic fascial appearance (p <0.001) were independent prognostic parameters for histological evidence of NF.

**Conclusion:**

Intraoperative tissue evaluation by an experienced surgeon is the most important diagnostic tool in identifying necrotizing fasciitis. An intraoperative Gram stain is an independent prognostic tool and therefore its use can be recommended especially in case of clinical uncertainty.

## 1. Introduction

Necrotizing fasciitis (NF) is a rare but lethal disease. NF is characterized by primary severe inflammation and necrosis of the fascia, leading to subsequent inflammation and necrosis overlying subcutaneous fat, muscle, and skin. NF manifests as local redness without sharp margins, swelling, warmth, and pain. Later, the infection spread rapidly along the fascial planes and subcutaneous tissue, leading to acute systemic toxicity [1]. Up to 50% of patients develop sepsis or respiratory failure and almost one-third of patients develop acute kidney failure [2], leading to a 90-day mortality rate of 18%, despite adequate medical intervention and aggressive surgical therapy [3].

Fortunately, the incidence of NF is relatively low, affecting 1.99–4.8 per 100 000 people/year [3, 4]. This disease does not seem to have any predilection for age but occurs slightly more often in male patients [2, 5]. NF usually affects the extremities and perineum and less frequently the trunk and cervical region. The etiology of this disease remains unclear. In some cases, a history of trauma, insect bites, scratches, or skin abrasion is present. In many others, the portal of entry is not identified [6].

Pre-existing conditions that result in immunosuppression, such as advanced age, chronic renal failure, peripheral vascular disease, diabetes mellitus, and drug misuse are risk factors for NF [6]. A large spectrum of species ranging from aerobic or facultative anaerobic species to polymicrobial infections has been implicated in the pathogenesis of NF [7].

Despite the advances in the medical field, our understanding of the pathogenesis and etiology of NF remains poor. The presenting signs and symptoms are usually subtle and non-specific, making the identification of NF challenging [6]. Early clinical recognition of NF and differentiation from other soft tissue infections like cellulitis is crucial since delayed initiation of therapy is a determinant factor for high mortality [8]. Adjunct tools like imaging studies [9, 10], gram stain, frozen section biopsy [11], and laboratory scores [12, 13] have been previously used for the discrimination between NF and other soft tissue infections with varying results, limitations, and availability.

Radical tissue resection remains the mainstay of therapy, which sometimes requires abdominal or thoracic exploration or even limb amputation to damage-control this devastating disease [5]. Furthermore, surgery results usually in large wound defects, which should preferably be treated at tertiary centers, that can offer complex reconstructive surgeries and rehabilitation [14].

In this analysis, we evaluated the outcome of patients with necrotizing fasciitis treated at a tertiary reference center in northern Germany to define the clinical parameters that make the identification of NF more precise and thus enable adequate therapy at an early stage in the course of the disease.

## 2. Method and materials

### 2.1. Patient selection

This retrospective study was conducted at the University Medical Center Hamburg-Eppendorf, a regional tertiary referral centre in northern Germany. All the patients who underwent surgical exploration for suspected NF between March 2010 and August 2017 were included. This study was approved by the ethic committee of Hamburg Chamber of Physicians (Nr. 2023–300303). Patient consent was waived by the ethics committee due to the retrospective nature of the study.

NF was suspected if patients presented with signs of severe local soft tissue inflammation and systemic toxicity. The indication for surgical exploration was made by an experienced surgeon. Intraoperative histopathological and microbiological analysis was performed to confirm or rule out the diagnosis. The soft tissue infection was considered necrotizing fasciitis only after the histological confirmation. Histological criteria included: (i) microscopic fascial necrosis, (ii) presence of PMNs or presence of gram-positive cocci in the fascia or deep skin layers, and (iii) the presence of thrombi in the fascial vessels.

Patient demographics, preoperative investigations, operative interventions, blood tests, and postoperative complications were recorded. The Clavien Dindo classification was used to grade the severity of surgical complication. The grades range from I to V. The grades are defined as follows, Grade I: Any deviation from the normal postoperative course without the need for pharmacological treatment or surgical, endoscopic, or radiological interventions. Grade II: Requiring pharmacological treatment with drugs other than those allowed for Grade I complications. Grade III: Requiring surgical, endoscopic, or radiological intervention. Grade IV: Life-threatening complications requiring intensive care unit management. Grade V: Death of a patient [15].

The intraoperative findings and surgical impressions were retrospectively recorded by a review of surgery reports. This study was approved by the institutional review board at the University of Hamburg.

### 2.2. Statistical analysis

Statistical analysis was carried out using *IBM SPSS ver*. *24 (Armonk*, *N*.*Y*., *USA)*. The t-test was used for continuous variables. The χ2 test and Fischer’s test were employed for categorical variables. A *p* ≤ of 0.05 was considered significant. Multivariate analysis to detect parameters associated with the presence of necrotizing fasciitis was performed using logistic regression. Multivariate analysis to detect parameters associated with mortality was carried out using logistic regression. The results were presented as hazard ratios with 95% CI. All comparisons were two-tailed. A *p* of less than 0.05 was considered statistically significant.

## 3. Results

### 3.1. Patient characteristics and perioperative outcome

We retrospectively identified eighty-eight (88) patients, who underwent surgical exploration for suspected necrotizing fasciitis. 55 out of 88 (63%) patients were males. The mean age was 56 ± 16 years. The soft tissue infection was in the lower extremities in 48 patients, in the thoracocervical region in 18 patients, and in the perineum and abdomen in 22 patients. The suspicion of NF was histologically confirmed in 59 of 88 patients.

Out of the 88 patients, 37 (27 NF and 10 without NF) patients required preoperative ICU (intensive care unit) treatment. 30 (23 NF and 7 without NF) patients required preoperative vasopressor therapy. 19 (16 with NF and 3 without NF) patients were preoperatively intubated. Patients with NF had a longer hospital length of stay of 41.39 (±50) vs. 21 (±19) days when compared to patients without NF (p = 0.01). The ICU length of stay was also longer in patients with NF 13 (±19) vs. 4 (±4) (p = 0.001) compared to patients without NF (Table 1).

**Table 1:** Impact of the clinical and perioperative findings and their impact on the presence of necrotizing fasciitis.

Variable	Necrotising Fasciitis ^a^	No Necrotising Fasciitis ^b^	P
**Clinical findings**			
Age	55 ± 14	57± 18	
Female sex	40%	41%	0.598
Redness	91%	96%	0.369
Fever	38%	24%	0.262
Pain	80%	62%	0.130
Swelling	89%	96%	0.255
Identifiable portal of entry	21%	18%	0.729
**Laboratory**			
Leucocytosis ≥ 13 x 10^9^ cells/L	51%	52%	0.938
Creatinine ≥ 2 mg/dl	51%	35%	0.147
Lactate ≥ 2.8 mmol/L	44%	14%	0.005
CRP ≥ 200 mg/L	61%	59%	0.829
**Perioperative Findings**			
Preoperative ICU Admission	49%	36%	0.178
Preoperative Intubation	29%	11%	0.060
Preoperative Vasopressor	41%	25%	0.147
Intraoperative surgical impression NF	95%	3%	<0.001
Positive Gram stain	43%	15%	0.028

*P* Indicates significance according to the χ2 test when comparing patients with and without necrotizing fasciitis. (%); percentage.

^a^; percentage in patients with NF.

^b^; percentage in patients without NF.

SD; standard deviation. ICU; intensive care unit. UTI; urinary tract infection

Typical intraoperative signs of necrotizing fasciitis like the presence of necrotic tissue, lack of tissue resistance, loose fascia on blunt dissection, and foul ‘dishwater’ pus were present in 56 patients. In all these patients a radical necrosectomy and fasciotomy were performed and the histology of NF was confirmed. Seven patients had equivocal clinical signs, in three patients a radical necrosectomy and fasciotomy were performed after obtaining a positive gram stain result. In the other four patients, a radical necrosectomy and fasciotomy were spared due to negative gram stain results. The diagnosis of NF was confirmed in 86% of the cases of positive gram stains.

In five patients a laparotomy was performed due to intraabdominal extension of limb NF. Two patients required a sternotomy due to mediastinitis and intrathoracic extension of cervical NF. Eight patients required limb amputation due to NF.

We report a high incidence of postoperative complications in our cohort. Severe postoperative complications (Clavien-Dindo III-V) were present in 80 (91%) patients, in 56 of them with NF. Of the 88 patients, 26 patients (29%) died in same the hospital period, 19 (22%) of whom had necrotizing fasciitis. The incidence of cardiovascular, pulmonary, renal, and hepatic failure is presented in (Table 2). In 18 patients (14 with NF, 4 without NF) laparotomy was performed after the initial exploration due to abdominal compartment syndrome or colonic ischemia.

**Table 2:** Postoperative complications in necrotizing fasciitis.

**Variables**	Mean/n (SD)/(%)		P
**Hospital length of stay**			0.01
*Necrotizing fasciitis*	41 (±50)		
*No Necrotising fasciitis*	22 (±19)		
**ICU length of stay**			0.001
*Necrotizing fasciitis*	13 (±19)		
*No Necrotising fasciitis*	4 (±4)		
**Mortality**			0.436
*Total deaths*	30%		
*Necrotizing fasciitis*	32%		
*No Necrotising fasciitis*	24%		
**Clavien-Dindo Classification**			
*0*	0		
*I*	1%		
*II*	8%		
*III*	21%		
*IV*	41%		
*V*	30%		
**Complications**	**Necrotising Fasciitis** (%)*	**No Necrotising fasciitis** (%)**	**P**
Acute Heart Failure	42%	29%	0.404
Acute Kidney Failure	71%	66%	0.588
Acute Liver Failure	35%	14%	0.042
Pneumonia	39%	21%	0.086
UTI	9%	10%	0.793
New onset Arrhythmia	36%	17%	0.173
GI-Bleeding	3%	3%	0.989
Catheter induced Infections	7%	3%	0.520
Abdominal emergency	24%	14%	0.277
Bleeding	5%	3%	0.599

*P* Indicates significance according to the χ2 test when comparing patients with and without necrotizing fasciitis. (%) percentage.

*; percentage in patients with NF.

**; percentage in patients without NF.

SD; standard deviation. ICU; intensive care unit. UTI; urinary tract infection

Regarding in-hospital perioperative mortality, our study shows that preoperative vasopressor use (*p* < 0.001), preoperative elevated lactate ≥ 2.8 mmol/dl (*p* < 0.001), preoperative ICU admission (*p* < 0.001), and preoperative intubation-need (*p* = 0.003) were associated with increased in-hospital patient mortality (Table 3). However, the multivariate analysis did not identify any independent prognostic parameters regarding in-hospital perioperative mortality.

**Table 3:** Univariate analysis of perioperative variables associated with mortality.

Variables	Mortality (%)	P
**Postoperative complications**		
Acute heart failure	55%	<0.001
Acute kidney failure	39%	0.002
Acute liver failure	50%	0.011
Pneumonie	17%	0.076
Urinary tract infection	13%	0.260
New-onset arrythmia	54%	0.003
Postoperative acute abdomen	56%	0.007
**Preoperative laboratory Findings**		
Leucocytosis > 13x10^9^ cells/L	29%	0.890
Creatinin > 2 mg/dl	38%	0.135
Lactate > 2.8	53%	< 0.001
CRP > 200 mg/L	28%	0.753
**Perioperative Findings**		
Preopeative ICU Admission	51%	< 0.001
Preoperative Intubation	58%	0.003
Preoperative vasopressor therapy	57%	< 0.001
Positive Gram stain	27%	0.600
Intraoperative surgical impression of NF	32%	0.436
Re-Operation	23%	0.065

*p* Indicates significance according to the χ2 test when comparing variables to mortality. (%) percentage in deceased.

### 3.2. Prediction of necrotizing fasciitis

The most common presenting clinical signs and symptoms in patients with NF were redness (91%), swelling (89%), and pain (80%). Fever was only present in (38%) of the patients. An identifiable portal of entry was present in 21% of the cases (Table 1).

In the preoperative blood tests, the detection of leucocytosis (≥13 x 10^9^ cells/L), elevated CRP (≥200 mg/L) or elevated creatinine (>2 mg/dL) were not associated with the presence of NF. Only elevated lactate (>2.8 mmol/L) at presentation was associated with the presence of NF in the histology (p = 0.005).

Preoperative CT or MRI was performed in 66 of the 88 patients. In 42 patients, imaging studies were suggestive of NF. In 33 patients, histology confirmed the diagnosis of NF. In 9 patients, the imaging studies showed false-positive results. Overall, the results of imaging studies were associated with the presence of NF in the definitive histology (p = 0.015).

Intraoperative Gram stain was performed in 44 patients with NF and 20 patients without NF. A positive Gram stain was present in 19 (43%) patients with NF and 3 patients without NF. Gram stain showed a positive predictive value of 86% and a negative predictive value of 40%. Overall, Gram stain was associated with the presence of NF in the definitive histology (p 0.028).

Multivariate analysis using logistic regression for factors associated with necrotizing fasciitis confirmed that liver failure (p = 0.019), sepsis (p = 0.011), positive Gram stain (p = 0.032), and macroscopic fascial appearance (p< 0.001) are independent prognostic parameters for histological evidence of NF (Table 4).

**Table 4:** Multivariate analysis of factors associated with necrotizing fasciitis.

Variable	Multivariate Analysis		
	HR	95%CI	P
Liver failure	1,917	0.674–3.160	0.019
Sepsis	2.075	0.231–3.919	0.011
Lactate, *≥2*.*8 vs <2*.*8 mmol/dl*	1.416	0.440–2.392	0.131
Radiological evidence of NF	1.107	0.564–1.65	0.272
Positive Gram stain	2.832	1.604–4.060	0.032
Surgical Impression of NF	9.567	8.132–11.002	<0.001

*p* Indicates significance according to regression analysis comparing the specified variables. CI indicates a 95% confidence interval; HR indicates hazard ratio

The ROC curve analysis of multiple perioperative parameters showed that the surgeon’s intraoperative impression of fascial appearance was the best prognostic factor for necrotizing fasciitis (AUC 0.977; (95% CI 0.924–1.000); p < 0.001).

## 4. Discussion

Necrotizing fasciitis is the most severe form of skin and soft tissue infection [16]. Its presentation is variable and usually challenging. Due to the initial subtle signs and symptoms, NF can be underestimated at presentation. This is followed by rapid clinical deterioration and extension of the disease, which contributes to the disease’s high morbidity and mortality [3, 17].

In this study, we report a high incidence of morbidity and mortality associated with NF (Table 2). The mortality rate was 22% in patients with NF. 91% of all patients with severe soft tissue infection and 96% of patients with NF had postoperative complications (Clavien-Dindo ≥III). However, these results come from a tertiary referral center, with a high number of referred critically ill and immune-compromised patients. Moreover, this mortality rate is consistent with other case series [18–20].

NF should be suspected in patients with classical signs of soft tissue inflammation (*rubor*, *dolor*, *calor*, *tumor)* when additional signs of systemic illness like fever, confusion, and hemodynamic instability are present [21].

However, in the absence of clinical factors, like a clear portal of entry, fever, local erythema, or in cases where pain can be falsely attributed to other factors like trauma or recent surgery, as well as in intubated patients, where history and examination are limited, the presence of adjunct diagnostic tools might help guide therapy [21, 22].

The most used inflammatory laboratory parameters, like elevated WBC and CRP, are also present in other soft tissue infections like cellulitis, making these parameters non-specific in the diagnosis of NF. In our cohort, leucocytosis (≥13 x 10^9^ cells/L) and elevated CRP (≥200 mg/L) were not associated with NF (*p* = 0.938 and 0.829). Only elevated Lactate ≥ 2.8 at presentation was associated with the histological evidence of NF and with perioperative mortality (*p = 0*.*005 and p < 0*.*001*, *respectively)*. This goes in line with other publications in the literature [23, 24]. Furthermore, in 2004, the Laboratory Risk Indicator for Necrotizing Infection (LRINEC) was proposed as a tool to distinguish NF from other soft tissue infections. According to Wong et al. a score > 8 is associated with a high risk for NF (75%). However, the following studies showed conflicting results [25–27] and a recent meta-analysis showed that LRINEC scores of ≥6 and ≥8 were associated with a low sensitivity of 68% and 40% [28]. Therefore the current guidelines do not recommend the use of the LRINEC score to rule out NF [17].

Till now, there is no consensus on the best imaging modality for the management of NF. Due to the low sensitivity of plain radiography, its use in NF is obsolete. It detects the presence of gas along the fascial planes only in 25% of cases [29]. On the other hand, a CT scan is more informative, especially for lower limb infection near the trunk to detect intraabdominal extension of the infection, and to possibly plan a simultaneous laparotomy [30]. According to Fernando et al., the CT scan was associated with a high sensitivity of 88.5% and specificity of 93.3% compared to plain radiography. Making CT scan an important adjunct tool in the diagnosis of NF. Furthermore, robust data about the utility of magnetic resonance imaging (MRI) in necrotizing fasciitis is still not available [28]. Nevertheless, existing evidence suggests a high sensitivity for MRI, which might even overestimate the degree of inflammation [31, 32]. In our cohort, the results of imaging studies were significantly associated with the presence of NF in the definitive histology in univariate analysis, but no significance was found in multivariate regression analysis.

However, in case of clinical uncertainty and equivocal diagnostic results, A low threshold for surgical exploration is justified and even necessary since it is the only reliable way to confirm the diagnosis or rule out NF [21]. Intraoperative findings like the presence of necrotic tissue, lack of tissue resistance, loose fascia on blunt dissection, and foul ‘dishwater’ pus are suggestive of NF and justify a radical tissue resection and debridement [33].

But in the presence of ambivalent intraoperative findings, like the presence of isolated subcutaneous edema, intraoperative decision-making becomes once again difficult provided that the surgeon has to rely only on the intraoperative impression when forming his judgment. In these cases we recommend combining intraoperative macroscopic appearance with the results of Gram stain, imaging studies, and preoperative lactate, to help differentiate between the various forms of soft tissue infections since their treatment strategies are fundamentally different.

For example, In cases of soft tissue cellulitis, intravenous antibiotics suffice. In case of abscess formation, incision and drainage are required. However, in the presence of NF, radical necrosectomy and fasciotomy should be performed until healthy resection margins are reached.

Considering this, Hietbrink et al. proposed an algorithm, in which intraoperative biopsies are taken for frozen section and Gram stain. If either one is positive, a radical debridement of the area follows [11]. In our cohort, seven patients had equivocal intraoperative findings, in three patients a radical tissue resection was performed after obtaining a positive gram stain test. In the other four patients, a radical resection was spared due to negative Gram stain results. In all these cases, the gram stain findings correlated with the histology. However, in one patient with a positive Gram stain, a radical resection was abandoned due to macroscopic viable fascia, in this case, the postoperative course was uneventful. This patient was discharged without further interventions.

In our cohort, Gram stain had a positive predictive value of (83%) and was significantly associated with histological evidence of NF in the univariate (p 0.028) and in the multivariate analysis (p 0.032). Nevertheless, Gram stain was only performed in 64 (73%) patients, due to limitations in its availability, which highlights a major drawback of this diagnostic tool. Furthermore, our results showed that macroscopic fascial appearance was a reliable parameter for the diagnosis of NF in multiple regression analysis (HR 9.5, CI95% 8.1–11, p < 0.001). With these observations, we can recommend the use of intraoperative Gram stain during surgical exploration for early identification of patients requiring extensive surgical necrosectomy and fasciotomy, especially in equivocal clinical scenarios.

Several limitations should be considered when interpreting our results. Due to the retrospective design of the study and the lack of treatment protocols, an inconsistency in patient selection and surgical management by different surgeons likely existed. However, we report a detailed outcome evaluation for a large number of patients undergoing surgical exploration for suspected NF at a tertiary referral center.

In our opinion, necrotizing fasciitis is a visual diagnosis since there is no laboratory test or imaging study that can replace surgical exploration to establish its diagnosis. However, due to the low incidence of NF, surgical expertise is variable. For this reason, we identified important objective parameters to confirm the diagnosis of NF. In addition to the macroscopic assessment of the fascia, Gram staining, CT/MRI and preoperative lactate value determination are relevant parameters for identifying NF and should be therefore routinely performed in cases of suspected NF.

## 5. Conclusion

Intraoperative evaluation of the fascial macroscopic appearance by an experienced surgeon is the most important diagnostic tool in treating necrotizing fasciitis. An intraoperative Gram stain was found to be an independent prognostic tool and therefore its use can be recommended in case of clinical uncertainty.

## Supporting information

S1 Table Original dataset. Cohort data used for the primary analysis.
(PDF)
