# Supplementary material for: Prognostic factors for intraoperative detection of necrotizing fasciitis in severe soft tissue infections
Source: PLoS One. 2023 May 3;18(5):e0285048. doi: 10.1371/journal.pone.0285048 (PMC10156062; doi:10.1371/journal.pone.0285048)
Supplement: S1 Table — Cohort data used for the primary analysis. (PDF) [file pone.0285048.s001.pdf]

No	Hospital Day:	Sex	Mortality	Morbidy	Clavien Dind	acute heart f	acute kidney
1	1	2	1	1	5	1	1
2	77	1	0	1	4	1	1
3	22	1	0	1	4	0	0
4	60	1	0	1	4	0	0
5	3	1	0	1	4	0	1
6	300	1	0	1	4	1	1
7	36	1	1	1	5	0	1
8	8	1	1	0	5	1	1
9	6	2	0	1	4	0	0
10	69	1	0	1	4	1	1
11	19	2	0	1	4	0	0
12	84	1	0	1	4	1	1
13	45	2	0	1	4	1	1
14	40	2	0	1	4	0	0
15	34	1	0	1	4	0	1
16	183	1	1	0	5	1	0
17	20	1	1	0	5	1	1
18	0	1	1	1	5	1	1
19	39	1	0	1	4	1	1
20	38	1	0	1	3	1	1
21	23	1	0	2	4	0	0
22	2	2	1	1	5	0	1
23	99	1	0	1	4	1	1
24	44	2	0	1	4	0	1
25	27	2	0	1	3	0	1
26	19	1	0	1	3	0	0
27	74	2	0	1	3	0	0
28	2	2	1	1	5	0	1
29	2	1	1	1	5	1	1
30	4	1	1	1	5	0	1
31	42	2	0	0	3	0	1
32	63	2	0	1	4	1	1
33	69	1	0	1	4	1	1
34	24	2	0	0	4	0	0
35	1	1	1	1	5	1	1
36	27	1	0	1	0	0	0
37	47	1	0	1	4	3	1
38	42	1	0	1	4	0	0
39	11	1	0	0	2	0	0
40	69	2	0	1	4	0	1
41	84	2	0	1	4	0	1
42	44	2	0	1	4	0	0
43	33	2	0	1	4	0	1
44	44	1	0	1	4	1	1
45	4	2	1	1	5	1	1
46	3	1	1	1	5	1	1
47	55	1	0	1	3	0	0
48	20	1	0	1	3	0	1
49	31	2	0	1	3	0	1

50	15	1	1	1	5	0	0
51	91	1	0	1	2	1	1
52	1	1	1	1	5	0	1
53	4	2	1	1	5	1	1
54	1	1	1	1	5	1	1
55	3	2	1	0	5	0	1
56	4	1	1	1	5	1	1
57	76	2	0	1	4	0	0
58	3	1	0	1	3	0	0
59	145	1	0	1	4	0	1
60	19	2	0	1	3		1
61	36	1	0	1	4	0	1
62	50	1	0	1	4	0	1
63	29	2	0	1	4	0	1
64	13	1	0	1	3	0	1
65	3	1	1	1	5	1	1
66	4	2	0	0	2	0	0
67	20	1	0	1	4	0	0
68	6	2	0	1	1	0	0
69	2	1	1	1	5	1	1
70	10	1	0	1	3	0	0
71	2	1	0	1	3	0	1
72	17	1	1	1	5	1	1
73	1	2	1	0	5	1	1
74	11	1	0	1	2	0	0
75	27	1	0	1	3	0	1
76	16	2	1	1	5	1	1
77	47	2	0	1	4	1	1
78	19	1	0	0	2	0	0
79	20	2	0	1	3	0	1
80	6	1	0	0	2	0	0
81	62	1	0	1	4	0	1
82	28	1	0	1	3	0	0
83	25	1	0	0	3	0	0
84	16	2	0	0	3	0	1
85	44	2	1	1	5	1	1
86	4	2	1	1	5	1	1
87	15	1	1	1	5	0	1
88	83	1	0	1	4	0	1

Pneumonia	UTI	Liver failure	new arrythm	SSI	GI Bleeding	catheter assc	Abdominal e
0	0	1	1	1	0	0	0
1	0	1	1	1	0	0	0
0	0	0	0	1	0	0	0
1	0	0	0	0	0	0	0
0	0	1	0	1	0	0	0
0	0	1	0	1	0	0	0
0	0	0	0	1	0	0	0
0	0	0	1	1	0	0	0
0	0	0	0	0	0	0	0
1	1	0	0	1	0	1	0
0	0	0	0	1	0	0	0
1	0	0	1	1	0	0	1
0	0	0	2	2	0	0	1
1	0	0	1	1	0	0	1
1	0	0	0	1	0	0	0
1	0	0	0	1	0	0	0
0	1	1	0	1	0	1	1
0	0	1	1	1	0	0	0
1	0	1	0	1	1	0	0
1	0	0	0	1	0	0	0
1	0	0	1	1	0	0	0
1	0	0	0	1	0	0	0
1	0	0	0	1	0	0	0
1	1	0	0	0	0	0	0
0	0	0	0	0	0	0	0
0	0	0	0	1	0	0	0
1	0	0	0	0	0	0	0
0	0	1	0	1	0	0	1
0	0	1	0	1	0	0	0
1	0	0	0	0	0	0	1
0	1	1	1	1	0	0	1
1	1	0	1	1	0	0	0
1	0	0	1	1	0	0	0
0	0	0	0	1	0	0	0
0	0	0	1	0	0	0	1
0	0	0	0	1	0	0	0
0	0	0	0	0	0	0	0
0	0	0	0	1	0	0	0
0	0	0	0	1	0	0	0
1	0	0	1	1	0	0	1
1	0	0	0	1	0	1	1
0	0	0	0	1	0	0	0
0	0	0	0	1	0	0	0
1	0	1	1	1	0	1	1
0	0	1	1	1	0	0	1
0	0	1	1	1	0	0	0
0	0	0	0	0	0	0	0
0	0	1	0	1	0	0	0
0	0	0	1	1	0	0	0

0	0	0	0	0	0	0	0
1	0	1	0	0	0	0	0
0	0	0	0	0	0	0	0
1	0	1	1	1	0	0	0
0	0	1	1	0	0	0	0
0	0	1	1	1	0	0	1
0	0	1	1	1	0	0	1
0	0	0	0	1	0	0	0
0	0	0	0	0	0	0	0
1	0	0	0	1	1	0	0
0	1	0	1	1	0	0	0
0	0	0	0	1	0	0	0
0	0	1	0	1	0	0	0
1	0	0	0	1	0	0	0
0	0	0	0	0	0	1	0
1	0	1	0	1	0	0	1
0	0	0	0	0	0	0	0
0	0	0	0	1	0	0	0
0	0	0	0	1	0	0	0
0	0	0	0	1	0	0	0
0	0	0	0	0	0	0	0
0	1	0	0	0	0	0	0
0	0	0	0	1	0	0	1
0	0	0	1	1	0	0	0
0	0	0	0	0	0	0	0
1	0	0	0	1	0	0	0
1	0	0	1	0	0	0	0
0	1	0	0	1	0	0	0
0	0	0	0	0	0	0	0
0	0	0	0	1	0	0	0
0	0	0	0	1	0	0	0
1	0	0	0	1	0	0	0
0	0	0	0	0	0	0	0
0	0	1	0	1	0	0	0
1	0	0	0	0	0	0	0
0	0	0	1	0	0	0	0
0	0	0	1	0	0	0	1
0	0	1	0	0	0	0	1
0	0	1	0	1	1	0	0

Bleeding pos'	Sepsis	ICU-Dauer	CRP	CRP>200	WBC	L>13	Creatinin
0	1	1	103	0	0,3	0	1,95
0	1	1	300	1	5	0	4,2
0	0	1	359	1	19,7	1	1
0	1	6	389	1	7,9	0	1,05
1	1	91	266	1	12,3	0	2,7
0	1	10	266	1	12,3	0	2,67
0	1	4	139	0	0,2	0	2
0	1	2	387	1	43	1	2,4
0	1	3	116	0	28,7	1	1,8
0	1	66	333	1	26,8	1	2,9
0	1	0	104	0	8,2	0	0,6
0	1	33	12	0	7	0	2,5
0	1	10	258	1	23	1	1,9
0	1	32	383	1	15,9	1	0,7
0	1	36	211	1	4,9	0	3,9
0	0	2	200	0	15	1	1
0	1	20	543	1	7,2	0	4,2
0	1	0	183	0	44,2	1	4,1
0	1	23	322	1	11,3	0	6,33
0	0	0	68	0	9	0	1,4
0	1	3	285	1	1,8	0	1,1
0	1	2	324	1	23,4	1	2,5
0	1	27	336	1	8	0	3,9
0	1	3	403	1	7,9	0	3,5
1	1	3	144	0	18,2	1	1,2
0	1	2	114	0	11,4	0	1,46
0	1	11	371	1	67	1	0,75
0	1	1	266	1	6,9	0	3,57
0	1	2	221	1	1,3	0	3,17
0	1	1	263	1	1,8	0	2,75
0	1	36	136	0	17,4	1	2,09
0	1	64	100	0	26,4	1	1,9
0	1	43	129	0	28,6	1	3
0	0	6	223	1	26,6	1	0,64
0	1	0	232	1	8	0	2,1
0	0	0	175	0	24,5	1	0,73
0	1	10	132	0	8,8	0	2,2
0	0	3	273	1	20,2	1	0,99
0	0	0	130	0	16,4	1	0,8
0	1	45	293	1	28,4	1	2,8
0	1	15	107	0	12	0	4,1
0	0	0	104	0	11,8	0	0,6
0	1	2	216	1	22	1	1,8
0	1	14	353	1	9	0	2,4
0	0	5	59	0	14	1	3,7
0	1	3	265	1	17,3	1	8,7
0	1	12	229	1	8,7	0	6,8
0	1	4	215	1	13,6	1	1,35
0	1	13	200	0	14,6	1	1,56

0	0	14	205	1	3,6	0	1,2
0	1	14	279	1	0,9	0	1,3
0	0	1	193	0	21,4	1	6,92
0	1	4	408	1	9,5	0	1,2
0	1	1	141	0	40,4	1	1,9
0	1	2	115	0	5,3	0	1,9
0	1	3	222	1	65	1	0,89
0	1	2	228	1	18	1	0,8
0	0	3	89	0	16,9	1	0,78
0	1	25	436	1	16,2	1	2,8
0	0	4	294	1	40	1	1
0	0	0	101	0	13,1	1	1
1	1	39	266	1	12,3	0	2,7
1	0	0	199	0	5,6	0	1,25
0	1	1	384	1	19	1	3,2
0	1	3	426	1	1,2	0	2,8
0	0	0	29	0	4	0	0,6
0	0	2	218	1	20	1	0,7
0	0	2	225	1	17	1	0,9
0	1	2	59	0	5	0	6,6
0	0	1	15	0	5,4	0	1
0	1	2	209	1	12	0	2
0	1	5	10	0	14,7	1	1,2
0	1	1	220	1	17,8	1	1,5
0	0	2	267	1	12,6	0	1
0	1	5	315	1	10,2	0	3,1
0	1	3	300	1	14,3	1	1,2
0	1	10	187	0	10	0	1,3
0	0	1	8	0	15,3	1	0,68
0	1	6	172	0	13,2	1	3,3
0	0	0	39	0	5,2	0	0,73
0	1	19	312	1	12	0	4,8
0	0	2	313	1	32	1	0,81
0	1	5	367	1	15,7	1	1,01
0	1	6	438	1	18,5	1	1,2
0	1	7	330	1	8,4	0	2,5
0	1	3	237	1	18,9	1	2,3
0	1	13	132	0	11,2	0	1
0	1	7	288	1	41,4	1	2,9

Crea>2	Lactate	Lac>2,8	Pre-OP ICU	Pre-OP intub	Pre-Op Nora	Redness	Fever
0	2,8	0	0	0	0	1	0
1	8,1	1	0	0	0	0	1
0		0	0	0	0	1	1
0		0	0	0	0	1	1
1	9,4	1	0	0	0	1	1
1	9,4	1	0	0	0	1	1
1	0,9	0	1	0	0	1	1
1	4,9	1	0	0	0	1	0
0	3,4	1			1	1	1
1	1,5	0	1	1	1	1	0
0		0	0	0	0	1	0
1	9,3	1	0	0	0	1	1
0		0	0	0	0	1	
0	2,6	0	0	0	0	1	0
1	7,1	1	1	0	1	1	0
0	2,2	0					
1	7,9	1	1	0	1	1	1
1	18	1	1	1	1	1	
1		0	1	0	0	1	1
0		0				1	0
0		0	0	0	0	1	0
1	0,8	0	0	0	0	1	1
1	4,4	1	1	0	1	1	0
1	1,6	0	0	0	0	1	
0	4,5	1	0	0	0	1	0
0	2,7	0	0	0	0	1	0
0	2,3	0	1	0	0	1	0
1	3,9	1	1	1	1	1	1
1	5,6	1	1	0	1	1	
1	0,3	0	1	1	1	1	0
1	8,3	1	1	1	1	1	
0	1,1	0	1	1	1	1	0
1	2,3	0	0	0	0	0	0
0	1,4	0	1	1	1	0	0
1	30	1	1	1	1		
0	1,2	0	0	0	0	1	
1	2,2	0	0	0	0	0	1
0	1,4	0	0	0	0	1	0
0	1,1	0	0	0	0	1	0
1	2,1	0	1	1	1	1	
1	4,6	1	0	0	0	1	
0		0	0	0	0	1	1
0	3,4	1					
1	2,1	0	1	1	0	1	0
1	17	1	1	0	1	1	0
1	14	1	1	1	1	1	1
1	1,5	0	0	0	0	1	0
0	1,7	0	0	0	0	1	1
0	1,1	0	0	0	0	1	1

0	3,3	1	1	1	1	1	0
0	3,9	1	1	0	1	1	1
1	1,7	0	1	0	1	1	0
0	3,8	1	1	1	1	1	
0	7,2	1	1	0	0	1	0
0	12,5	1	1	1	1	0	0
0	8,8	1	1	1	1	1	
0	1,3	0	0	0	0	1	
0		0	0	0	0	1	0
1	1,8	0	1	1	1	1	0
0	1,3	0	0	0	0	1	1
0	2	0	0	0	0	1	
1	9,4	1	0	0	0	1	0
0		0	0	0	0	1	1
1	2,3	0	0	0	0	1	0
1	2,5	0	1	0	1	1	0
0	0,7	0	0	0	0	1	0
0	1	0	1	0	0	1	0
0		0					
1	2,1	0	0	0	0	1	0
0	0,7	0	0	0	0	1	0
1	0,9	0	0	0	0	1	0
0	9,6	1	0	0	0	1	0
0	5,3	1	1	1	1	1	0
0	2	0	1	0	0	1	
1	6,6	1	1	0	1	1	
0	0,7	0	1	0	1	1	0
0	1,7	0	0	0	0	1	1
0		0	0	0	0	1	
1	0,6	0	1	1	1	1	0
0		0	0	0	0	1	0
1	0,8	0	0	0	0	1	1
0	1,9	0	0	0	0	1	0
0	1	0	1	0	0	1	
0	1,3	0	0	0	0	0	
1	1,5	0	0	0	0	1	0
1	1,2	0	1	0	1	1	0
0	6,3	1	1	1	1	1	
1	2,5	0	0	0	0	1	0

Pain	Swelling	Portal of ent	Pre-OP CT	Pre-OP MRT	Diagnosis Fa	Cito-Mibi	Gram Stain r
1	1	0	1	0	1	1	1
1	1	0	1	0	1	1	1
0	1	0	0	1	1	1	1
1	1	0	1	0	1	1	1
1	1	0	0	1	0	1	1
1	1	0	0	1	0	1	1
1	0	0	1	0	1	1	1
0	1	0	0	1	1	1	1
1	1	0	0	1	0	1	1
0	1	0	0	1	1	1	1
1	1	0	0	1	1	1	1
1	1	0	0	0	0	1	1
1	1	0	0	0	0	1	1
1	1	0	1	0	1	1	1
1	1	0	0	0	0	1	1
	0	0	0	0	0	1	1
1	1	0	1	0	0	1	1
	1	1	1	0	1	1	1
1	1	1	1	0	0	1	0
1	1	0	0	1	1	1	0
1	1	0	0	1	0	1	0
	1	0	0	1	1	1	0
0	1	0	0	0	0	1	0
1	1	0	0	1	1	1	0
1	1	0	0	1	1	1	0
1	1	0	1	0	1	1	0
0	1	1	1	0	0	1	0
1	1	0	1	0	0	1	0
	1	0	1	0	1	1	0
0	1	0	1	0	1	1	0
	1	0	1	0	1	1	0
0	1	0	1	0	1	1	0
1	1	0	0	0	0	1	0
0	0	0	1	0	1	1	0
	0	0	1	0	1	1	0
	1	0	1	0	1	1	0
1	0	0	1	0	1	1	0
1	1	1	0	0	0	1	0
1	0	0	1	0	1	1	0
	1	1	1	0	0	1	0
	1	1	1	0	0	1	0
1	1	0	0	1	0	1	0
		0	0	0	0	1	0
		0	0	1	1	1	0
1	1	0	1	0	0	1	0
1	1	1	0	1	1	1	0
1	1	0	0	1	1	1	0
1	1	0	0	0	0	1	0
1	1	0	1	0	0	1	0

		1	1	0	1	1	0
1	1	0	0	0	0	1	0
1	1	0	0	0	1	1	0
	1	1	0	0	1	1	0
1	1	0	1	0	1	1	0
1	0	0	1	0	1	1	0
	1	1	1	0	0	1	0
	1	1	0	1	0	1	0
0	1	0	0	1	1	1	0
1	1	0	0	0	0	1	0
1	1	1	0	0	0	1	0
	1	0	0	0	0	1	0
1	1	1	0	1	0	1	1
1	1	1	0	1	1	1	1
1	1	0	0	1	1	1	1
0	1	0	0	0	0	1	1
0	1	0	0	1	1	1	0
0	1	0	0	1	1	1	0
			0	0	0	1	0
1	1	0	1	0	0	1	0
1	1	1	0	0	0	1	0
1	1	0	0	1	1	1	0
1	1	1	1	1	0	1	0
0	1	0	1	0	1	1	0
	1	0	0	0	0	1	0
	1	0	1	1	0	1	0
1	1	0	0	1	0	1	0
0	1	1	0	0	0	1	0
	1	0	1	0	0	1	0
0	1	0	0	0	0	1	0
0	1	0	1	0	1	1	0
1	1	0	1	0	1	1	0
1	1	0	0	1	1	1	0
	1	0	0	1	0	1	0
	0	0	1	0	0	1	0
1	1	0	0	0	0	1	0
1	1	0	0	0	0	1	0
	1	0	0	0	0	1	0
0	1	0	0	1	0	1	0

Macroscopic	Resection	Ergebnis MIB	Keim Nachw	HISTOplusMI	Diagnose Fas	Re-OP
1	1	0	1	1	1	0
1	1	1	1	1	1	1
1	1	1	1	1	1	1
1	1	1	1	1	1	1
1	1	1	1	1	1	0
1	1	1	1	1	1	1
1	1	1	1	1	1	0
1	1	0	1	1	1	1
1	1	1	1	1	1	1
1	1	1	1	1	1	1
1	1	1	1	1	1	0
1	1	1	1	1	1	1
1	1	1	1	1	1	1
1	1	1	1	1	1	1
1	1	1	1	1	1	0
1	1	1	1	1	1	0
1	1	0	1	1	1	1
1	1	1	1	1	1	0
1	1	1	1	1	1	1
1	1	1	1	1	1	0
1	1	1	1	1	0	1
1	1	0	1	1	1	1
1	1	1	1	1	1	1
1	1	0	1	1	1	1
1	1	1	1	1	1	1
1	1	1	1	1	1	1
1	1	0	1	1	1	1
1	1	1	1	1	1	1
1	1	1	1	1	1	0
1	1	0	1	1	1	1
1	1	1	1	1	1	1
1	1	0	1	1	1	0
1	1	1	1	1	1	1
1	1	0	1	1	1	1
1	1	1	1	1	1	0
1	1	0	1	1	1	1
1	1	0	1	1	1	0
1	1	0	1	1	1	1
1	1	0	0	1	1	1
1	1	1	1	1	1	1
1	1	0	1	1	1	1
1	1	0	0	0	0	1
1	1	1	1	1	1	0
1	1	1	1	1	1	1
1	1	1	1	1	1	1
1	1	1	1	1	1	0
1	1	1	1	1	1	1
1	1	1	1	1	1	1
1	1	0	1	1	1	0

1	1	0	1	1	1	1
1	1	0	1	1	1	0
1	1	0	1	1	1	0
1	1	1	1	1	1	0
1	1	0	0	1	1	0
1	1	1	1	1	1	1
1	1	1	1	1	1	1
1	1	1	1	1	1	1
1	1	0	1	1	1	1
1	1	0	1	1	1	1
0	0	0	1	0	0	1
0	0	0	0	0	0	1
0	0	1	1	1	1	1
0	0	0	1	0	0	0
0	0	0	1	0	0	0
0	0	1	1	1	0	0
0	0	0	1	0	0	0
0	0	1	1	1	0	1
0	0	0	0	0	0	0
0	0	0	1	0	0	0
0	0	0	0	0	0	1
0	0	0	1	0	0	0
0	0	1	1	1	0	1
0	0	1	1	1	0	0
0	0	0	0	0	0	0
0	0	1	1	1	0	0
0	0	1	1	1	0	0
0	0	1	1	1	0	1
0	0	1	1	1	0	1
0	0	0	0	1	1	0
0	0	0	0	0	0	0
0	0	1	1	1	1	1
0	0	0	1	0	0	0
0	0	0	0	0	0	1
0	0	0	1	0	0	0
0	0	0	0	0	0	0
0	0	0	1	1	1	1
0	0	0	1	0	0	1
0	0	1	1	1	0	1

FDFasziitis 1ja 0 nein
1
1
1
1
1
1
1
1
1
1
1
1
1
1
1
1
1
1
1
1
1
1
1
1
1
1
1
1
1
1
1
1
1
1
1
1
1
1
1
1
1
0
1
1
1
1
1
1
1

1
1
1
1
1
1
1
1
1
1
0
0
1
0
0
0
0
0
0
0
0
0
0
0
0
0
0
0
0
0
0
0
0
0
0
0
0
0
0
